# Effects of Sequential Fermentation with *Saccharomyces bayanus* and *Lactobacillus brevis* on the Metabolite Composition and Antioxidant Activity of Chinese Yam Juice

**DOI:** 10.3390/foods15061055

**Published:** 2026-03-17

**Authors:** Aroosa Mushtaq, Zhoumei Huang, Xiangning Ma, Jun Sun, Chen Ma, Fang Chen

**Affiliations:** 1 College of Food Science and Nutritional Engineering, National Engineering Research Centre for Fruit and Vegetable Processing, China Agricultural University, Beijing 100083, China; aroosamushtaq62@gmail.com (A.M.); xiangningma95@gmail.com (X.M.); sjun8585@163.com (J.S.); machen21@cau.edu.cn (C.M.); 2 College of Food Science and Engineering, Wuhan Polytechnic University, Wuhan 430023, China

**Keywords:** Chinese yam, sequential fermentation, lactic acid bacteria, metabolic profiles, antioxidant activity

## Abstract

Chinese yam (*Dioscorea opposita* Thunb.) is a nutrient-rich tuber with recognized health benefits, yet its application in beverage products remains limited due to processing and formulation challenges. In this study, a sequential fermentation strategy was adopted, using *Saccharomyces bayanus* followed by *Lactobacillus brevis* to enhance microbial viability and metabolic activity in Chinese yam juice. Samples were collected as an unfermented control (CY), yeast-fermented juice (SP), and sequentially fermented juice (LB). Microbial analysis showed that sequential fermentation supported high LAB viability, reaching 8.92 log CFU/mL in LB, accompanied by a progressive decrease in pH from 5.67 (CY) to 4.27 (LB). Untargeted LC-MS/MS metabolomics identified 1442 metabolites and revealed distinct shifts in the metabolic composition of CY, SP, and LB, indicating stage-dependent modifications of metabolic pathways. Targeted analyses confirmed substantial depletion of sucrose and maltose during fermentation, while trehalose accumulated from undetectable levels in CY to 5.23 mg/g in SP and 7.49 mg/g in LB. Organic acid profiling demonstrated marked increases in lactic and succinic acids, consistent with microbial carbohydrate metabolism. Total phenolic and flavonoid contents increased by 58% and 30%, respectively, while antioxidant capacity (DPPH, ABTS, and FRAP) improved by up to 120% after sequential fermentation. The final fermented beverage (LB) contained a low ethanol concentration of 0.8% (*v*/*v*). Sensory evaluation indicated that sequential fermentation improved the overall flavor, aroma, and acceptability of the Chinese yam juice. These findings demonstrate that sequential fermentation with *S. bayanus* and *L. brevis* effectively enhances the bioactive composition and antioxidant potential of Chinese yam juice, supporting its development as a functional fermented beverage.

## 1. Introduction

Chinese yam (*Dioscorea opposita* Thunb.) is a nutrient-dense tuber widely consumed in East Asia and recognized in the Chinese Pharmacopoeia for its health-promoting properties. It is particularly rich in bioactive compounds, including polysaccharides, polyphenols, and steroidal saponins, which have been associated with antioxidant, antidiabetic, and immunomodulatory activities [1,2]. Owing to its favorable phytochemical profile, Chinese yam has attracted increasing interest as a raw material for the development of functional foods and beverages [3,4]. However, its application in beverage formulations remains limited due to inherent technological challenges, for example, high viscosity arising from mucilage and starch, which compromises colloidal stability, sensory acceptability, and the bioaccessibility of bioactive compounds [5,6]. Fermentation represents an effective biotechnological strategy to overcome these limitations while simultaneously enhancing nutritional quality and generating novel bioactive metabolites [7,8]. Evidence suggests that adding probiotic cultures and bioactive compounds derived from fermented waste to animal feed can enhance the productivity of species such as goats and swine. This approach offers an environmentally sound alternative to traditional feed supplements. This aligns with circular economic models by reintegrating organic by-products into agricultural systems and reducing reliance on externally sourced additives [9,10,11].

Among fermentative microorganisms, lactic acid bacteria (LAB) are widely used for their acidification capacity and ability to produce functional metabolites. In our previous work, *Lactobacillus brevis* was identified as a suitable starter culture for Chinese yam fermentation, demonstrating favorable fermentation performance and enhanced bioactive compound production [12]. Nevertheless, fermentation relying solely on LAB may result in products with limited sensory complexity, particularly when applied to starch-rich matrices. Sequential fermentation incorporating yeast before LAB inoculation offers a promising alternative. Yeasts such as *Saccharomyces bayanus* contribute to flavor development and matrix modification by consuming oxygen and releasing metabolites that facilitate subsequent LAB growth [13]. Importantly, *S. bayanus* exhibits a strong capacity for trehalose accumulation [14]. The complementary metabolic functions of *S. bayanus* (trehalose enrichment) and *L. brevis* (acidification and bioactive compound production) provide a biochemical basis for their combined, sequential application in the development of functional fermented beverages [15]. Although sequential fermentation strategies have been explored in other plant-based substrates [13,15], their application to Chinese yam juice, particularly involving a yeast inactivation step to prevent ongoing yeast metabolism from competing with LAB, has not been reported.

The objectives of this study are to develop a sequential fermentation of Chinese yam juice with *S. bayanus* and *L. brevis*, and to comprehensively evaluate the changes in physicochemical properties, antioxidant activity, and targeted and non-targeted metabolomic profiles of the Chinese yam juice.

## 2. Materials and Methods

### 2.1. Materials

Chinese yam (*Dioscorea opposita* Thunb.) used in this study was purchased from a local market in Beijing, China. The microbial cultures, *Saccharomyces bayanus* (yeast, internal code SP) and *Lactobacillus brevis* (LB-17), were provided by the National Engineering Research Center for Fruits and Vegetables Processing at China Agricultural University, Beijing, China. The strains were maintained and routinely subcultured under standard laboratory conditions before use. Culture media, including YPD and MRS broths, were purchased from Aoboxing Biotechnology Co., Ltd. (Beijing, China). Kits for analyzing total phenolic content (TPC), total flavonoid content (TFC), and ferric reducing antioxidant power (FRAP) were obtained from SolarBio Science & Technology Co., Ltd. (Beijing, China). All other analytical-grade chemicals were sourced from Sinopharm Chemical Reagent Co., Ltd. (Shanghai, China).

### 2.2. Chinese Yam Juice Preparation

Chinese yam juice was prepared using a modified version of the protocol established by Chen et al. [15]. First, fresh yam tubers were thoroughly washed, peeled, and rinsed with distilled water. They were then cut into pieces approximately 2 cm in size and briefly immersed in boiling water for five minutes to inactivate naturally occurring enzymes. After cooling, the blanched yam was combined with distilled water at a weight-to-volume ratio of 1:1.5 and homogenized using a commercial food processor (Longxing Chemical Machinery Group Co., Linyi, Shandong, China) for 45 s at 18,000 rpm to obtain a smooth slurry. The slurry was then filtered through sterile muslin cloth to separate the juice. The resulting filtrate was collected as the raw yam juice for subsequent processing.

### 2.3. Microbial Culture and Inoculum Preparation

*Saccharomyces bayanus* and *Lactobacillus brevis* were prepared for fermentation following a revised method described by Guan et al. [16]. The yeast culture was revived from a −80 °C glycerol stock, propagated through three consecutive cycles in yeast extract–peptone–dextrose (YPD) broth, and incubated aerobically at 30 °C for 24 h with shaking at 180 rpm. Similarly, *L. brevis* was subcultured three times in de Man, Rogosa, and Sharpe (MRS) broth at 37 °C under static conditions for 24 h. Following growth, microbial cells from both cultures were harvested by centrifugation (4000× *g*, 10 min, 4 °C), rinsed three times with sterile distilled water, and resuspended to a standardized inoculum density (OD600 = 1.0 ± 0.05).

### 2.4. Production of Fermented Chinese Yam Juice

After juicing, the homogenate was treated with α-amylase (0.08% *w*/*v*) to reduce starch content and enhance transparency. The mixture was heated to 90 °C for 30 min, held without heating for an additional 30 min, and then brought to 85 °C for 10 min to deactivate the enzyme and eliminate potential microbial contaminants. Once cooled, the clarified juice was transferred under aseptic conditions to sterilized fermentation containers. Sequential fermentation was performed according to the procedure described by Cheng et al. [13]. Pasteurized yam juice was first inoculated with *S. bayanus* (5% *v*/*v*) and fermented aerobically at 30 °C for 24 h. The resulting product was heat-treated at 65 °C for 15 min to stop yeast activity, cooled to 37 °C, and then inoculated with *L. brevis* (3% *v*/*v*). The second fermentation proceeded anaerobically in sealed vessels at 37 °C for 12 h. Three sample types were collected for comparison: unfermented yam juice (CY), juice after yeast fermentation (SP), and the final product after sequential fermentation with *L. brevis* (LB). All samples were flash-frozen and stored at −80 °C for subsequent analysis.

### 2.5. Microbial Enumeration and pH Measurement

The viable cell concentrations of *S. bayanus* were quantified by plating on YPD agar, and *Lactobacillus brevis* was enumerated using MRS agar, following a dilution and plating method adapted from prior fermentation studies [17]. Fermented juice samples were serially diluted in sterile saline (0.9% NaCl) before plating on the appropriate media. Incubation settings varied by microorganism: yeast plates were incubated aerobically at 30 °C for 48 h, while *L. brevis* was cultured anaerobically at 37 °C for the same period. Colonies were counted only on plates containing 30–300 colonies. All counts were performed visually, and colony morphology was assessed for consistency. The results are reported as Log CFU/mL. The pH of the samples was measured using a calibrated digital pH meter (INESA Analytical Instrument Co., Ltd., Shanghai, China).

### 2.6. Ethanol Content Determination

The level of ethanol (% *v*/*v*) of the fermented Chinese yam juice samples (CY, SP, LB) was determined using a portable probe-type Anton Paar alcohol meter (Snap 41, Anton Paar GmbH, Graz, Austria). Before analysis, samples were centrifuged at 8000× *g* for 10 min to remove suspended solids. Subsequently, the measurement probe was inserted into about 5 mL of the supernatant, and the alcohol content was recorded. The instrument was calibrated using distilled water before measurement.

### 2.7. Untargeted Metabolomics Analysis

#### 2.7.1. Juice Sample Preparation

To prepare the samples, 100 μL of juice samples (CY, SP, and LB) was added to 1.5 mL Eppendorf tubes and mixed with 400 μL of prechilled 80% methanol (*v*/*v*) to remove proteins. After vortexing, the samples were kept on ice for 5 min, then spun at 15,000× *g* for 20 min at 4 °C. The supernatant was collected, diluted with LC–MS–grade water until the methanol concentration reached 53%, and centrifuged again under the same conditions. The final supernatant was placed in LC vials for analysis. A pooled quality control (QC) sample was made by mixing equal amounts of all the sample extracts. This QC sample was injected at regular intervals during the analysis to ensure the instrument remained stable and provided consistent results.

#### 2.7.2. Chromatographic and Mass Spectrometric Analysis

Chromatographic separation and mass spectrometry were performed using an integrated ultra-high-performance liquid chromatography (UHPLC) system coupled with a high-resolution mass spectrometer (Thermo Fisher Scientific, Waltham, MA, USA). The UHPLC component utilized a reversed-phase C18 column (Hypersil GOLD, 100 × 2.1 mm, 1.9 µm particle size) maintained at 40 °C. A binary mobile phase system was employed, consisting of (A) 0.1% formic acid in water and (B) 0.1% formic acid in acetonitrile. The separation was achieved using the following gradient profile at a constant flow rate of 0.3 mL/min: an initial hold at 2% B for 2 min, followed by a linear increase to 95% B over 13 min (2–15 min), an isocratic hold at 95% B for 3 min (15–18 min), a rapid return to the initial conditions (18–18.1 min), and finally a 3.9 min re-equilibration period at 2% B (18.1–22 min). The injection volume was set to 2 µL. Mass spectrometric detection was performed using a hybrid quadrupole–Orbitrap analyzer (Q-Exactive HF) operating in both positive and negative ion modes via electrospray ionization. Full-scan mass spectra were acquired over a mass-to-charge (*m*/*z*) range of 70–1050, at a resolving power of 120,000. Data-dependent acquisition was configured to sequentially fragment the ten most abundant precursor ions from each full scan, acquiring tandem mass spectra (MS/MS) at three normalized collision energies (20, 40, and 60 eV) for each ion.

#### 2.7.3. Quality Control and Data Processing

To assess the reliability and consistency of the analytical platform, a pooled quality control (QC) sample was generated by mixing identical volumes of each experimental sample extract. This QC sample was injected at the start of the analytical sequence and then recurrently following every sixth test sample during the run. The data were analyzed using Compound Discoverer (Thermo Fisher Scientific, v. 3.3), which executed peak picking, retention time alignment, gap filling, and spectral deconvolution. Tentative compound identifications were made by matching the experimental MS/MS spectra to entries in the mzCloud and ChemSpider libraries using a mass error tolerance of 5 ppm for precursor ions and 10 ppm for product ions. For trustworthy downstream analysis, features showing a relative standard deviation (RSD) above 30% in repeated QC measurements were excluded from further statistical evaluation.

### 2.8. Physicochemical Properties Analysis

#### 2.8.1. Organic Acids Analysis

Organic acids in fermented Chinese yam juice were quantified by ultra-high-performance liquid chromatography coupled with multiple reaction monitoring tandem mass spectrometry (UHPLC-MRM-MS/MS) [18]. For extraction, 100 μL of the sample was mixed with 500 μL of 50% acetonitrile in water, vortexed for 1 min, sonicated in an ice bath for 10 min, and centrifuged at 12,000 rpm for 10 min at 4 °C. The resulting supernatant was filtered through a 0.22 μm PTFE membrane before analysis. Chromatographic separation was achieved using a Waters ACQUITY UPLC HSS T3 C18 column (2.1 × 100 mm, 1.7 μm) with water containing 0.1% formic acid (mobile phase A) and acetonitrile: methanol (3:1, *v*/*v*; mobile phase B) as eluents. The column temperature was set at 30 °C, and a 2 μL aliquot of each sample was injected. Detection was performed using an SCIEX QTRAP 6500+ triple quadrupole mass spectrometer operating in MRM mode, with optimized transitions for each analyte. Quantification was performed using external calibration curves generated from serially diluted standards. Metabolite concentrations were calculated based on extraction volume and sample mass, and results were expressed as ng per g of juice (wet weight). Analytical precision, expressed as relative standard deviation (RSD), and accuracy, determined by recovery, were evaluated using quality control (QC) samples. Recoveries ranged from 87% to 114%, with RSD values below 11%.

#### 2.8.2. Sugars Analysis

Sugar concentrations in Chinese yam juice were analyzed using a Thermo ICS-5000+ ion chromatography system (Thermo Fisher Scientific, Waltham, MA, USA) equipped with a pulsed amperometric (electrochemical) detector [19]. For sample preparation, 1 mL of each juice sample was diluted 5-fold with ultrapure water, vortexed to ensure homogeneity, centrifuged at 10,000× *g* for 5 min to remove particulates, and the supernatant was filtered through a 0.22 µm PTFE syringe filter prior to injection. The separation was performed on a CarboPac™ PA1 analytical column (250 mm × 4.0 mm) maintained at 30 °C, with a flow rate of 1.0 mL/min and an injection volume of 10 µL. A binary mobile phase system was used: eluent A, ultrapure water; eluent B, 100 mM sodium hydroxide. The following elution gradient was applied: 0 min, A phase/B phase (95:5, *v*/*v*); 12 min, A phase/B phase (90:10, *v*/*v*); 15 min, A phase/B phase (0:100, *v*/*v*); 25 min, A phase/B phase (0:100, *v*/*v*); 40 min, A phase/B phase (95:5, *v*/*v*); and 60 min, A phase/B phase (95:5, *v*/*v*). To minimize instrumental drift, all samples were analyzed in randomized order. Quality control (QC) samples, prepared by pooling equal aliquots of all study samples, were injected at regular intervals to monitor system stability. Chromeleon software (Thermo Fisher Scientific) facilitated baseline correction, peak identification, retention time alignment, and quantification. Sugar concentrations were determined using external standard curves and expressed as milligrams per gram of fresh weight (mg/g FW).

#### 2.8.3. Total Phenolic Content (TPC)

The phenolic content of the juice samples was determined using the Folin–Ciocalteu colorimetric method [20]. In each test, 0.5 mL of juice was mixed with 3.0 mL of distilled water and 0.2 mL of the Folin–Ciocalteu reagent. After allowing the mixture to stand for five minutes, 1.3 mL of a 10% (*w*/*v*) sodium carbonate solution was added. The samples were vortexed and kept in the dark at room temperature for two hours. Absorbance was measured at 765 nm using a Shimadzu UV-1800 spectrophotometer (Tokyo, Japan). A calibration curve was prepared using gallic acid standards (0–100 mg/L), and the results were reported as milligrams of gallic acid equivalents per milliliter of sample (mg GAE/mL). All analyses were performed in triplicate, with blank controls included in each experiment.

#### 2.8.4. Determination of Total Flavonoid Content (TFC)

The total flavonoid concentration was determined using an aluminum chloride-based colorimetric assay, following a procedure described by Dionice et al. [20], with adjustments. For analysis, 0.5 mL of the sample was diluted with distilled water to a final volume of 3.0 mL. Next, 150 µL of a 5% sodium nitrite solution was added, and the mixture was incubated for 6 min. Subsequently, 300 µL of 5% aluminum chloride solution was added. Following a 5 min reaction period, 1.0 mL of 1.0 M sodium hydroxide was used to stop the reaction. The absorbance was measured at 510 nm using a UV spectrophotometer. Quantification was based on a quercetin calibration curve (2–10 mg/L), and the flavonoid content was calculated and expressed as milligrams of quercetin equivalents per milliliter of juice (mg QE/mL). All measurements were conducted in triplicate.

### 2.9. Antioxidant Activity

#### 2.9.1. ABTS Free Radical Scavenging Assay

Antioxidant activity was assessed using an adapted ABTS radical cation decolorization method, based on the protocol described by Mantzourani et al. [21]. The ABTS^++^ solution was generated by allowing 7 mM ABTS to react with 2.45 mM potassium persulfate in distilled water. This mixture was kept in the dark at ambient temperature overnight (16 h). The resulting stock solution was diluted with ethanol until its absorbance reached 0.70 ± 0.02 at 734 nm. In the assay, test samples were introduced to the diluted ABTS solution, and the absorbance was measured after a 6 min reaction period using a UV-Vis spectrophotometer. Antioxidant capacity was expressed as the percentage of radical scavenging inhibition relative to the blank control. All analyses were performed in triplicate, and measurements were confined to the linear portion of the standard curve.

#### 2.9.2. DPPH Free Radical Scavenging Assay

The antioxidant capacity of the samples was evaluated using a DPPH (2,2-diphenyl-1-picrylhydrazyl) radical scavenging assay, adapted from Dionice et al. [20]. In this procedure, 1.0 mL of each sample extract was combined with 2.00 mL of a methanolic DPPH solution (0.10 mmol L^−1^). The mixture was briefly vortexed and incubated in the dark at ambient temperature for 30 min. Following incubation, the absorbance was measured at 517 nm using a UV-visible spectrophotometer. The percentage inhibition of DPPH radicals was determined using the following equation:

% scavenging activity = [(A_0_ − A_1_)/A_0_] × 100

where A_0_ is the absorbance of the control (DPPH solution without sample), and A_1_ is the absorbance of the sample. All measurements were performed in triplicate.

#### 2.9.3. Ferric Reducing Antioxidant Power (FRAP) Assay

The assay measured the total antioxidant capacity using the ferric reducing antioxidant power (FRAP) method. For each analysis, a fresh working FRAP solution was prepared by mixing acetate buffer (0.1 M, pH 3.6), 10 mM TPTZ solution, and 20 mM FeCl_3_·6H_2_O in a 10:1:1 volumetric ratio. In the assay, 0.1 mL of the prepared sample extract was combined with 2.9 mL of freshly prepared FRAP reagent and incubated at room temperature for 15 min. Absorbance was measured at 593 nm using a UV-visible spectrophotometer. The results were quantified by comparison with a standard calibration curve constructed using FeSO_4_ at concentrations ranging from 0 to 150 µM. The final antioxidant capacity was expressed as µmol Fe^2+^ equivalents per mL of sample.

### 2.10. Sensory Analysis

Sensory evaluation of Chinese yam juice samples (CY, SP, LB) was conducted to assess consumer acceptability of the sequentially fermented product, referring to the method of da Silva et al. [22]. A total of 20 untrained panelists (graduate students and staff members familiar with fermented beverages) participated in the evaluation. The samples were assessed for color, flavor (aroma), taste, and general acceptability using a 9-point hedonic scale where 1 = Dislike extremely, 5 = Neither like nor dislike, and 9 = Like extremely. The samples were served in randomized order and coded with three-digit random numbers to minimize bias. Approximately 30 mL of each sample was presented at room temperature (25 ± 2 °C) in transparent cups under controlled laboratory conditions. Drinking water was provided for palate cleansing between samples.

### 2.11. Statistical Analysis

Each measurement was performed in triplicate, and the results are expressed as the average ± standard deviation. To evaluate variation among sample sets, a one-factor analysis of variance (ANOVA) was used. When significant differences were detected, Duncan’s test was used for detailed group-wise comparisons (*p* < 0.05). SPSS (version 27.0, IBM Corp., Armonk, NY, USA) was used for statistical evaluations. In the metabolomics investigation, pattern recognition was achieved through principal component analysis (PCA) and orthogonal projections to latent structures discriminant analysis (OPLS-DA), which were executed in SIMCA-P (Version 16.0.2, Sartorius Stedim Data Analytics AB, Umeå, Sweden).

## 3. Results

### 3.1. Changes in Microbial Dynamics, pH Value, and Ethanol Content During Sequential Fermentation

**Figure 1:**
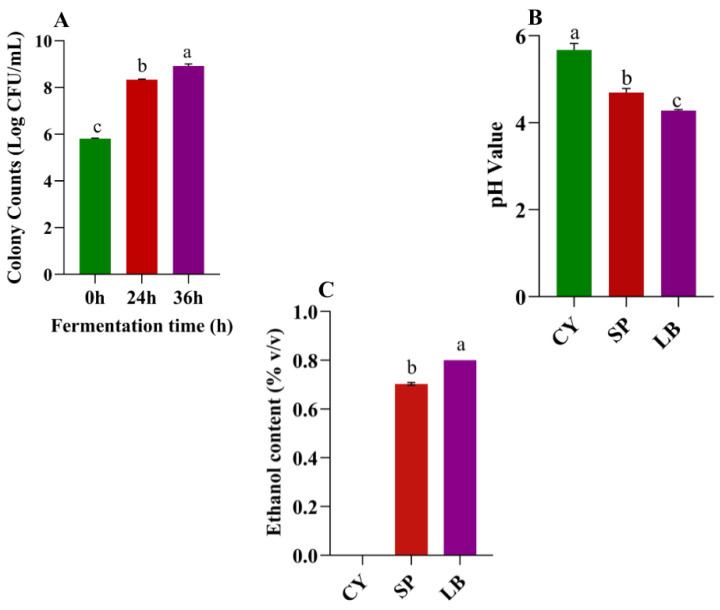
Microbial counts (**A**), pH (**B**), and ethanol content (**C**) of Chinese yam juice during fermentation. CY: control (0 h, non-fermented); SP: first-stage fermentation (24 h) with *Saccharomyces bayanus*; LB: second-stage fermentation (36 h) with *Lactobacillus brevis*. Data are means ± SD of triplicate readings. Letters indicate statistical differences (*p* < 0.05), (*p* < 0.0001) between samples based on one-way ANOVA.

The fermentation process influenced the microbial population. Compared to the unfermented juice (CY) (5.81 log CFU/mL), the microbial count reached 8.34 ± 0.02 log CFU/mL after the first fermentation stage by *S. bayanus* (SP, Figure 1A). Following heat inactivation of yeast, the CY was inoculated with *L. brevis* and started the second fermentation stage, through which the viable LAB count reached 8.92 ± 0.09 log CFU/mL in the final CY juice (LB). Additionally, a progressive decrease in pH was observed during the sequential fermentation (*p* < 0.05). After the first fermentation stage, the pH of the CY juice declined from 5.67 ± 0.15 to 4.69 ± 0.09, and further declined to 4.27 ± 0.03 after the second fermentation stage (Figure 1B). The ethanol content (% *v*/*v*) changed significantly during fermentation (Figure 1C). No detectable ethanol was observed in the control group (CY), whereas the SP and LB samples contained 0.70 ± 0.01 and 0.80 ± 0.01, respectively. The results demonstrate that ethanol was produced during fermentation. Although concentrations remained relatively low, they were significantly higher than those observed in CY (*p* < 0.0001).

### 3.2. Changes in the Metabolic Profile During Sequential Fermentation

**Figure 2:**
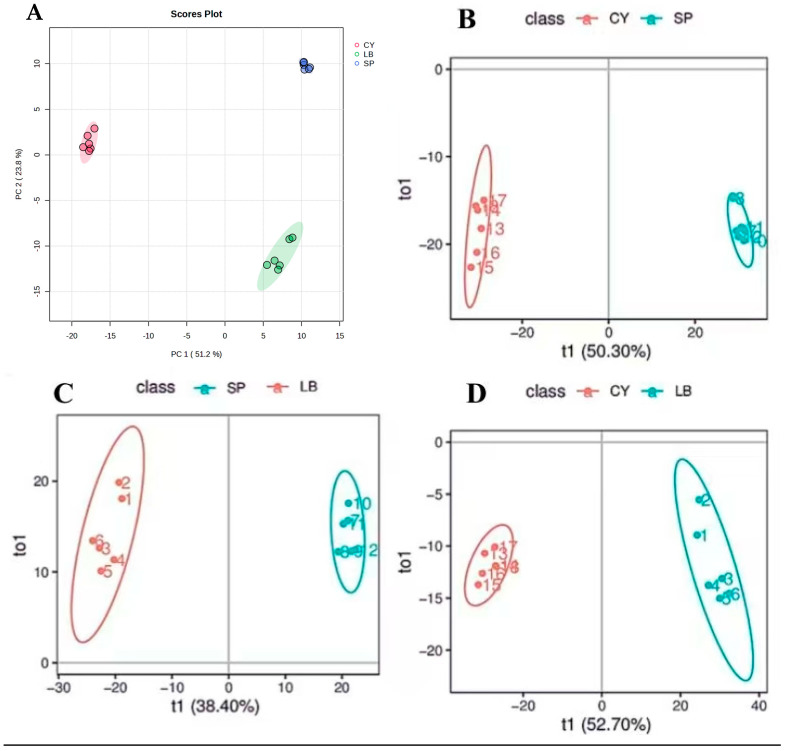

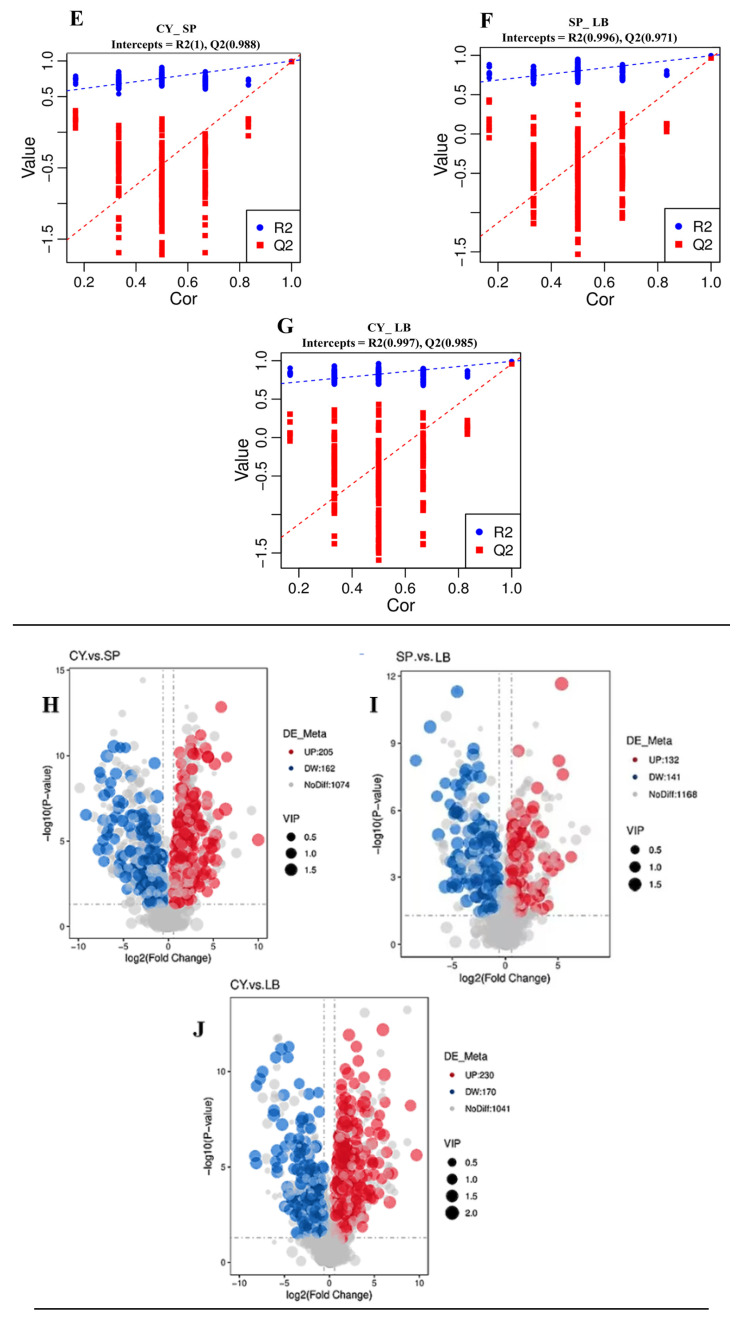

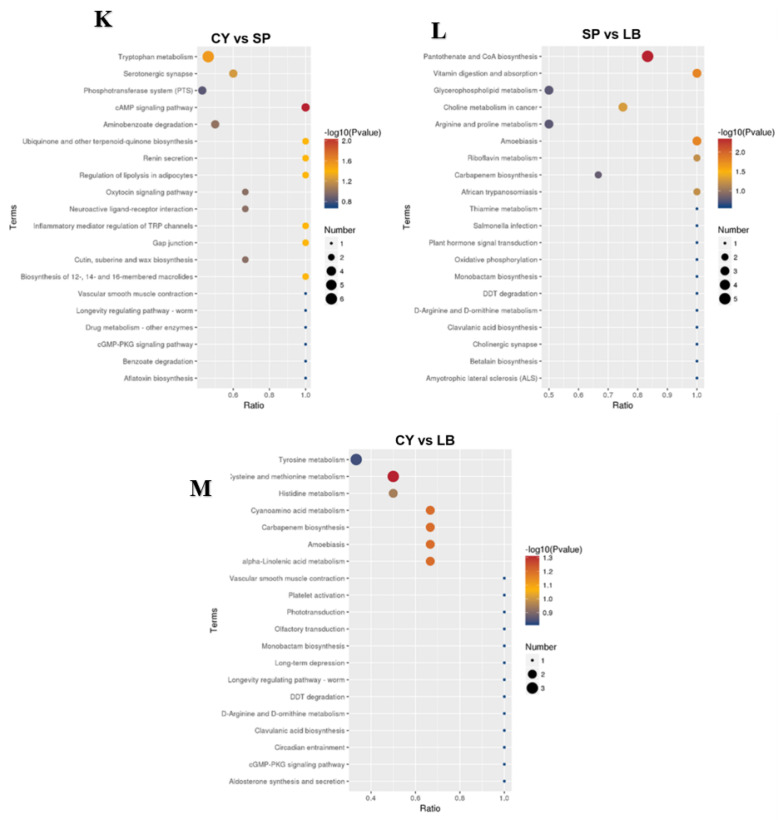
Metabolite changes in samples based on untargeted metabolomics analysis: PCA score plot illustrating the metabolic divergence induced by fermentation (**A**); orthogonal Partial least squares-discriminant analysis (OPLS-DA) score plots of metabolite profiles. Pairwise comparisons between fermentation stages: CY vs. SP (**B**); SP vs. LB (**C**); and CY vs. LB (**D**). The greater predictive variance in A and B reflects the larger metabolic shift from the unfermented state; C highlights the distinct metabolic outcome of bacterial co-culture compared to yeast fermentation alone. The intercept values of R^2^ and Q^2^ from the permutation test were determined in 2 (**E**–**G**). Volcano plot analysis of pairwise group comparisons with VIP-weighted significance (**H**–**J**); KEGG pathway enrichment analysis of differentially abundant metabolites (**K**–**M**).

To better understand the biochemical alterations induced by sequential fermentation with *Saccharomyces bayanus* and *Lactobacillus brevis*, an untargeted metabolomics analysis was conducted. In total, 1442 metabolites were detected across all samples, revealing extensive metabolic remodeling during fermentation (Figure S1A–C and Tables S1–S3). Principal component analysis (PCA) demonstrated clear and non-overlapping clustering among the unfermented control (CY), yeast-fermented (SP), and sequentially bacteria-fermented (LB) samples, accounting for 75.0% of the total variance (PC1: 51.2%; PC2: 23.8%) (Figure 2A). This unsupervised separation indicates pronounced stage-dependent metabolic divergence. Consistently, supervised orthogonal partial least squares-discriminant analysis (OPLS-DA) further confirmed complete discrimination among the three groups, highlighting distinct metabolic trajectories driven by yeast and lactic acid bacterial fermentation (Figure 2B–D). A 200-iteration permutation test was used to assess the OPLS-DA model’s validity and robustness. The intercept values of R^2^ and Q^2^ from the permutation test were determined to be (1.0, 0.988) for CY vs. SP, (0.996, 0.971) for SP vs. LB, and (0.997, 0.985) for CY vs. LB, as illustrated in Figure 2E–G. The R^2^ and Q^2^ values are represented by the blue squares and red circles, respectively. Crucially, all models Q^2^ regression lines showed a negative intercept and intersected the y-axis below zero, with all permuted Q^2^ values continuing to be far lower than the Q^2^ of the original model. Additionally, all models have exceptional strength of fit, as indicated by R^2^ intercept values that are close to 1.0. These findings validate the dependability of the discriminant metabolites by confirming that all three OPLS-DA models were strong, robust, and free from overfitting. Differential metabolite analysis using volcano plots (VIP > 1.0, *p* < 0.05) revealed substantial metabolic shifts across fermentation stages, with hundreds of metabolites significantly altered in each pairwise comparison (for example, 205 upregulated and 162 downregulated metabolites in CY vs. SP) (Figure 2H–J). Notably, the disaccharide Trehalose was consistently identified as a significantly upregulated metabolite across comparisons (CY vs. SP and SP vs. LB; *p* < 0.001), indicating that its accumulation may represent a characteristic metabolic signature of sequential fermentation.. Kyoto Encyclopedia of Genes and Genomes (KEGG) pathway enrichment analysis of the differentially abundant metabolites indicated that the complete fermentation process (CY, SP-LB) significantly affected carbohydrate metabolism pathways.

### 3.3. Targeted Quantification of Metabolite Accumulation During Sequential Fermentation

To validate the untargeted metabolomics findings, we performed targeted quantification of major sugars and organic acids in the sequentially fermented Chinese yam juice (Table 1 and Table 2). Eleven monosaccharides were quantified, revealing a clear pattern of carbohydrate depletion throughout the fermentation process (Table 1). Native sucrose and maltose were nearly exhausted after sequential fermentation, while glucose was largely consumed. Remarkably, trehalose, undetectable in the unfermented juice (CY), accumulated to 5.23 mg/g after yeast fermentation (SP) and further increased to 7.49 mg/g in the final product (LB). These findings confirm trehalose as a fermentation-specific metabolite, consistent with its identification in the untargeted metabolomics analysis.

Simultaneously, the profiles of 38 organic acids were markedly altered, reflecting active microbial metabolism. Succinic acid showed a significant sequential increase (*p* < 0.05) in comparison with the unfermented juice (Table 2). Additionally, lactic acid emerged as the dominant acid after the second fermentation stage (LB), exhibiting more than a sixfold increase relative to the first stage (SP). The dynamics of L-malic acid, however, were more complex and stage-dependent. Considering these stage-dependent acid changes, it is valuable to compare these results across different fermentation substrates. Across the sequential fermentation stages of Chinese yam juice, the observed accumulation of organic acids corresponds closely with the significant, stage-dependent decrease in total sugars (*p* < 0.05; Figure S2).

### 3.4. Impact of Sequential Fermentation on the Bioactive Components and Antioxidant Activity of Chinese Yam Juice

**Figure 3:**
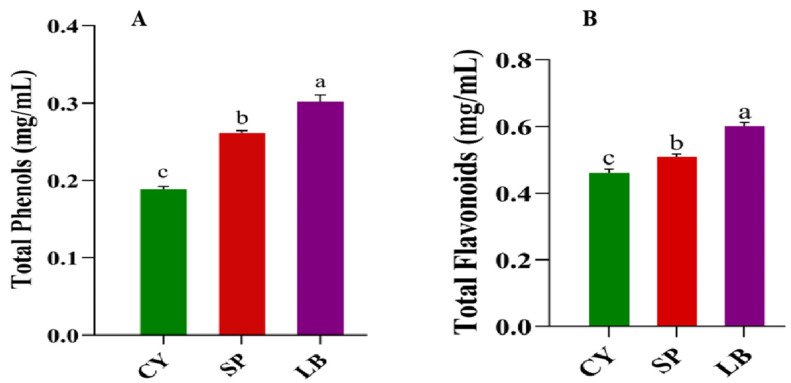
Changes in bioactive indicators of samples: Changes in the total phenol content (**A**) and total flavonoid content (**B**). CY is control; non-fermented Chinese yam juice, SP is fermented with *Saccharomyces bayanus*, and LB is sequentially fermented with *Lactobacillus brevis*. Data are means ± SD of triplicate readings, and Letters indicate statistical differences (*p* < 0.05) between samples based on one-way ANOVA.

**Figure 4:**
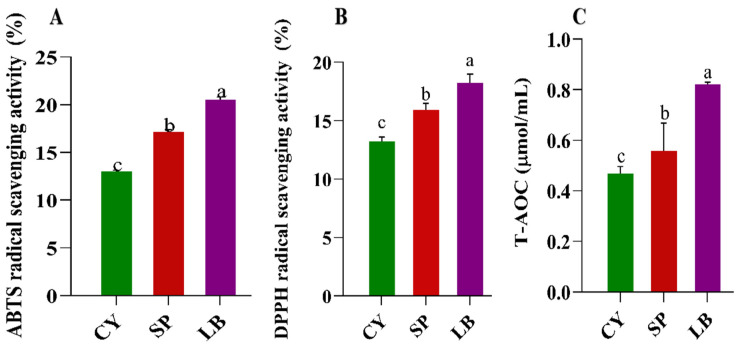
Changes in antioxidant activities: ABTS radical scavenging activity (%) (**A**); DPPH radical scavenging activity (%) (**B**); total antioxidant capacity (T-AOC) (µmol/mL) (**C**). Data are means ± SD of triplicate readings. Letters indicate statistical differences (*p* < 0.05) between samples based on one-way ANOVA.

The impact of sequential fermentation with *Saccharomyces bayanus* and *Lactobacillus brevis* on the functional components of Chinese yam juice was evaluated by measuring total phenolic content and total flavonoid content (Figure 3A,B). The total phenolic content (TPC) increased significantly throughout the fermentation process, reaching its highest level in the LB sample (Figure 3A). The change in total flavonoid content (TFC) also showed a similar trend (Figure 3B). Furthermore, to assess whether these compositional changes resulted in functional antioxidant effects, the radical scavenging capacity and total antioxidant potential of the juice were evaluated using DPPH and ABTS assays, as well as total antioxidant capacity (T-AOC) analysis. Fermentation significantly (*p* < 0.05) enhanced radical scavenging activity in a stage-dependent manner (Figure 4A–C). The first fermentation stage substantially increased antioxidant capacity, with rises of 61% and 73% in DPPH and ABTS scavenging activity, respectively, compared to the unfermented juice. The second fermentation stage (LB) further enhanced this effect, resulting in increases of 117% and 120% for DPPH and ABTS, respectively (Figure 4A,B). T-AOC provides an integrative assessment of overall redox potential. The T-AOC increased from 0.47 ± 0.02 in the unfermented juice to 0.65 ± 0.03 after the first fermentation (SP) and peaked at 0.82 ± 0.03 after the second fermentation (LB), with a 74% increase.

### 3.5. Sensory Attributes Analysis

**Figure 5:**
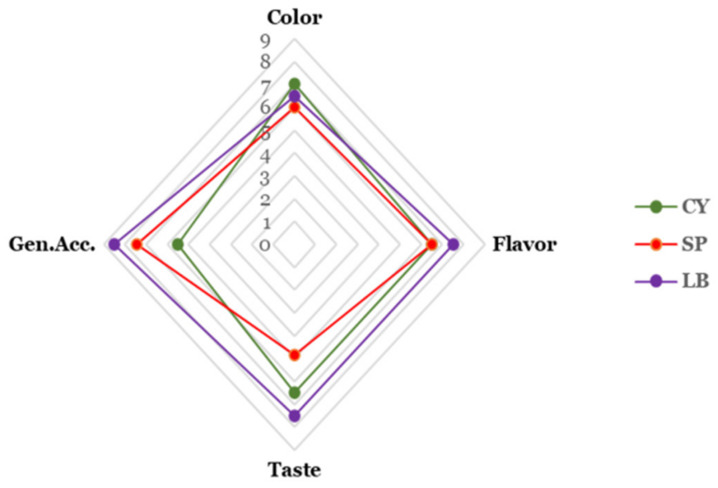
Radar chart comparing the median sensory scores of unfermented and fermented Chinese yam juice. CY, non-fermented control; SP, fermented with *Saccharomyces bayanus*; LB, sequentially fermented with *Lactobacillus brevis*.

The sensory acceptability of unfermented (CY), yeast-fermented (SP), and sequentially bacteria-fermented (LB) Chinese yam juice was evaluated using a 9-point hedonic scale, with results presented in Figure 5. Sequential fermentation significantly influenced the sensory profile of the juice, with marked differences observed across all attributes (color, flavor, taste, and overall acceptability) as fermentation progressed. The unfermented control (CY) received the highest score for color (7.2 ± 0.6), which was significantly higher (*p* < 0.05) than both fermented samples. In contrast, the sequentially fermented sample (LB) achieved the highest scores for flavor (7.5 ± 0.5), taste (7.1 ± 0.4), and overall acceptability (7.3 ± 0.5). The findings indicate that although fermentation may diminish color appeal, it significantly improves the flavor, taste, and overall acceptability of the juice.

### 3.6. Linking Metabolic Shifts to Functional Improvement

The integrated results from untargeted and targeted analyses show how sequential fermentation alters the composition of Chinese yam juice through distinct microbial metabolisms. *S. bayanus* initiates the transformation, consuming oxygen and simple sugars, and notably accumulating trehalose. The accumulation of trehalose, a known microbial stress protectant, is a distinctive biomarker of this specific fermentation process. While the direct contribution to in vitro antioxidant assays may be indirect, its presence indicates a successful and specific metabolic state. As a result, the improved antioxidant activity is probably linked to increased levels of phenolic and flavonoid antioxidants, and their release is catalyzed by the combined enzymatic activities of the two microorganisms.

## 4. Discussion

This study demonstrates the effective application of a sequential fermentation strategy utilizing *Saccharomyces bayanus* and *Lactobacillus brevis* to enhance the functional profile of Chinese yam juice. The process yielded high viable counts of lactic acid bacteria, a distinct metabolic signature, and significantly improved antioxidant properties, supporting its potential as a novel fermented functional beverage. The following discussion interprets these findings in the context of microbial metabolism and biotransformation, highlighting the synergistic role of the two fermentation stages.

The sequential fermentation process established a robust microbial environment conducive to both fermentation and a potentially probiotic carrier, exceeding the threshold of 10^7^ probiotic cells per gram for health-promoting claims for fermented products, according to the FAO and WHO guidelines [23]. The high final viability of *L. brevis* (8.92 log CFU/mL) meets or exceeds the threshold for probiotic efficacy in foods, a crucial attribute for functional beverages. The progressive decrease in pH from 5.67 to 4.27, primarily driven by lactic acid production in the second stage, serves multiple functions. This initial decrease in pH was consistent with findings from other *L. reuteri* fermentation processes [24]. The further decrease observed during the subsequent bacterial fermentation stage aligns with lactic acid bacteria (LAB)-mediated fermentation of medicinal plants using *Limosilactobacillus fermentum *14, *Limosilactobacillus reuteri *18, and *Lactiplantibacillus plantarum* [25]. This acidification not only ensures microbiological safety by inhibiting spoilage organisms but also contributes to the beverage’s sensory profile (tanginess) and may influence the stability and bioavailability of certain bioactive compounds. The initial pH drop after yeast fermentation likely created a selective environment favorable for the subsequent inoculation and dominance of acid-tolerant *L. brevis.* The observed low ethanol content (<1% *v*/*v*) in the fermented Chinese yam juice is permissible for natural fermentation processes and meets the requirement for non-alcoholic beverages in related regulations [26,27]. The specific metabolic imprint resulting from the yeast-LAB combination aligns with recent findings in starchy matrices. For instance, fermentation of pearl millet with *Saccharomyces cerevisiae* plus *Companilactobacillus paralimentarius*, similar to the sequence of *S. bayanus* and *L. brevis* used in this study, led to a significant improvement in nutritional properties compared with single-organism fermentations, highlighting the synergistic potential of such co-cultures in starch-rich substrates [28].

Untargeted metabolomics analysis revealed profound and stage-specific reprogramming of the juice chemical landscape. The clear separation of the control (CY), yeast-fermented (SP), and sequentially fermented (LB) samples in multivariate models underscores the unique metabolic imprint of each microbial phase. Targeted analyses provided a mechanistic explanation for these shifts, focusing on carbohydrate metabolism. The near-complete depletion of native sucrose and maltose, coupled with significant glucose consumption, confirms the efficient utilization of these carbon sources by both microorganisms, including starch and sucrose metabolism (Figure 2K–M) and pathways related to the biosynthesis of secondary metabolites [26]. The most striking finding was the accumulation of trehalose, a disaccharide absent in unfermented juice. Its concentration increased to 5.23 mg/g after yeast fermentation and further to 7.49 mg/g after bacterial fermentation, making it a signature metabolite of this specific process. Trehalose is a well-known microbial stress protectant, and its accumulation suggests a microbial response to fermentation environments. This may also contribute to the final product’s overall sensory and physicochemical properties. Its accumulation reveals trehalose’s role as a microbial stress protectant [29] and highlights its potential as a biomarker of microbial activity during fermentation.

The organic acid profile further illustrated the complementary roles of the microbes. The significant increase in succinic acid after the yeast stage aligns with *S. bayanus* activity via the TCA cycle. The subsequent dramatic rise in lactic acid is a direct result of the heterofermentative metabolism of *L. brevis*, solidifying its dominant role in the second stage. This increase is attributable to yeast activity via the tricarboxylic acid pathway during the second fermentation stage [30]. Similarly, the co-fermentation of pearl millet with specific yeast-LAB pairs significantly increased total phenols and released oligopeptides not present in the unfermented grain, an effect attributed to the combined metabolic activities breaking down the cereal matrix [28]. The dynamic behavior of L-malic acid, which initially increases and then decreases, highlights the complexity of acid metabolism, which can be influenced by microbial species, substrates, and redox conditions. This phenomenon is likely due to heterofermentative metabolism in *L. brevis* [31], and it is consistent with reports that malic acid metabolism depends on strain and fermentation conditions [32].

The most significant functional outcome of this bioprocessing strategy is the substantial improvement in bioactive content and antioxidant capacity. After the initial yeast fermentation, the concentration increased, which is consistent with previous reports of *L. reuteri*-mediated fermentations [33]. Sequential fermentation led to a 58% increase in total phenolic content (TPC) and a 30% increase in total flavonoid content (TFC). This enhancement is likely attributable to multiple factors: the enzymatic (glycosidase) activities of both microbes may have hydrolyzed bound phenolic compounds into their free, more extractable, and potentially more bioactive forms, and microbial metabolism may also have led to the bioconversion of existing compounds into new phenolic derivatives.

These compositional changes resulted in enhanced antioxidant performance, with DPPH and ABTS radical scavenging activities increasing by 117% and 120%, respectively, and FRAP increasing by 74%. The stage-wise improvement is consistent with a sequential effect in which the yeast stage modifies the substrate environment and the LAB stage strengthens acidification and biotransformation, producing the highest antioxidant response in the final product. This relationship reflects the progressive utilization of carbohydrates by *S. bayanus* and *L. brevis* to generate succinic, lactic, malic, and other organic acids [34]. The proposed role of the initial yeast stage in modifying the starchy yam matrix is consistent with reports in pearl millet, where yeast–LAB fermentations changed starch fractions and, in some co-culture systems, increased resistant starch content, indicating structural re-organization of starch polymers by microbial enzymes [35]. This mechanistic insight strengthens the argument that *S. bayanus* modifies the tuber matrix to facilitate subsequent *L. brevis* activity [35]. Flavonoid changes align with reports using *S. bourlai* [36], suggesting that microbial activity can enhance the release or bioconversion of these compounds [37]. According to Yang et al. [38] and Ozturk et al. [39], increases in antioxidant-related indices can reflect conversion of bound phenolics into free phenolic compounds and flavonoid aglycones under fermentation-derived enzymes, and related increases in total antioxidant capacity have been reported in fermented ginseng berry kombucha beverages and cereals [40,41,42]. However, the results demonstrated that the initial yeast stage likely modified the matrix and facilitated a more specialized action by LAB in the second stage [43].

The proposed mechanism underlying these improvements is synergistic and sequential. *Saccharomyces bayanus* acts as a primary modifier by consuming oxygen and simple sugars, altering the pH, and potentially initiating the breakdown of the yam matrix. This sets the stage for *L. brevis*, which performs a more specialized fermentation, efficiently producing lactic acid and further catalyzing the release and transformation of antioxidants. The unique metabolic fingerprint, including trehalose accumulation, is a biomarker of successful microbial succession. Subsequent *L. brevis* fermentation further utilizes carbohydrates to produce lactic acid and appears to facilitate the continued increase in trehalose and a significant rise in phenolic compounds [44]. From an application perspective, this study validates sequential fermentation as a powerful tool for adding value to Chinese yam. It moves beyond simple preservation or acidification, actively enhancing the juice’s health-promoting antioxidant profile and endowing it with probiotic viability. The resulting product aligns with the growing consumer demand for natural, fermented functional beverages. Future research should focus on scaling up the fermentation process, further optimizing sensory attributes at the industrial level, and validating health benefits through in vivo studies [45].

The sensory profile reveals differences in consumers’ perceptions of the samples. Differences were observed in the color, flavor, taste, and general acceptability of the samples. The difference observed in the color profile of the samples likely reflects the natural opacity and light coloration of raw yam juice, which diminished during processing, a common phenomenon in thermally or enzymatically treated vegetable juices. Panelists described the LB sample as having a pleasantly tangy, mildly acidic taste with a balanced fermented aroma, attributes likely imparted by the combined metabolic activities of *S. bayanus* and *L. brevis* during sequential fermentation. The organic acid profile of LB, particularly the increased lactic acid and modified malic acid content, likely contributes to this desirable sourness and flavor complexity [46].

The intermediate sample (SP), fermented only with yeast, received moderate scores across all attributes but was consistently rated lower than LB for taste, flavor, and overall acceptability, suggesting that the subsequent bacterial fermentation was essential for developing a well-rounded sensory profile. The sensory results indicate that while processing may reduce color appeal, the sequential fermentation strategy successfully produces a product with high consumer acceptability, driven by improved flavor and taste [47].

## 5. Conclusions

The sequential fermentation of Chinese yam (CY) juice using *Saccharomyces bayanus* followed by *Lactobacillus brevis* is an effective bioprocessing strategy that significantly reshapes the microbial, metabolic, and functional profiles of the CY juice. The process successfully establishes a high-density probiotic culture, achieving LAB counts exceeding the recommended threshold for health-promoting claims, while inducing a pronounced, stage-dependent acidification. Integrated metabolomics revealed an extensive biochemical shift, characterized by the selective depletion of native sugars and the distinctive, progressive accumulation of certain disaccharides, including trehalose. Furthermore, targeted quantification confirmed dynamic shifts in organic acid profiles, which align with the respective metabolic activities of yeast and lactic acid bacteria. Notably, these compositional transformations translated into substantial functional enhancement, as demonstrated by elevated total phenolic and flavonoid contents and a stage-wise improvement in antioxidant capacity. The final product of fermentation (LB) exhibited a low ethanol content of 0.8% (*v*/*v*). In addition, sensory evaluation showed that sequential fermentation improved the overall aroma and acceptability of Chinese yam juice. The synergistic, two-stage approach leveraged initial yeast activity to modify the substrate, thereby facilitating a more specialized bacterial metabolism in the subsequent stage. This work shows how controlled, sequential microbial transformation can unlock the bioactive potential of Chinese yam juice, primarily through the microbial-mediated release and bioconversion of antioxidant compounds, offering a promising avenue for developing novel, value-added functional beverages. Future research should comprehensively evaluate the stability of viable LAB counts and bioactive compounds during storage to better establish the functional potential and shelf-life characteristics of sequentially fermented Chinese yam juice.

## Figures and Tables

**Table 1:** Sugar contents in control (CY) and two sequentially fermented Chinese yam juice.

Sugars (mg/g)	Sample Name		
	CY	CY-SP	CY-SP-LB
Trehalose	ND	5.23 ± 0.89 ^b^	7.49 ± 0.19 ^a^
Fructose	ND	0.04 ± 0.003 ^a^	0.04 ± 0.002 ^a^
Arabinose	ND	ND	0.08 ± 0.002 ^a^
Galactose	ND	0.31 ± 0.02 ^a^	0.02 ± 0.02 ^b^
Glucose	53.25 ± 2.95 ^a^	12.70 ± 0.60 ^b^	0.92 ± 0.07 ^c^
Mannose	ND	0.08 ± 0.02 ^a^	0.01 ± 0.01 ^b^
Fucose	32.37 ± 4.12 ^a^	0.94± 0.067 ^b^	0.49 ± 0.11 ^b^
Sucrose	15.52 ± 1.73 ^a^	0.08 ± 0.02 ^b^	0.22 ± 0.17 ^b^
Raffinose	2.82 ± 0.27 ^a^	0.42 ± 0.02 ^b^	0.40 ± 0.01 ^b^
Stachyose	1.87 ± 0.48 ^a^	ND	0.17 ± 0.02 ^b^
Maltose	1.32 ± 0.38 ^a^	ND	0.25 ± 0.02 ^b^

Values are mean ± standard deviation (SD) of triplicate measurements. CY denotes non-fermented Chinese yam juice; SP, juice fermented with *Saccharomyces bayanus*; and LB, juice fermented with *Lactobacillus brevis*. ND indicates “Not Detected”. Different superscript letters denote statistically significant differences between samples (*p* < 0.05) as determined by one-way ANOVA.

**Table 2:** Organic acids content in control (CY) and two sequentially fermented Chinese yam juices (ng/g).

Serial No.	Compound Name	CY	CY-SP	CY-SP-LB
1	4-aminobutyric acid	41,439.00 ± 1721.25 ^a^	19,068.02 ± 2889.78 ^b^	45,315.93 ± 5796.01 ^a^
2	indolelactic acid	ND	1433.13 ± 166.12 ^a^	ND
3	kynurenic acid	ND	22,037.66 ± 1889.68 ^a^	13,304.57 ± 53.50 ^b^
4	2-indolecarboxylic acid	746.59 ± 97.79 ^a^	259.95 ± 24.05 ^b^	ND
5	pyroglutamic acid	424,194.50 ± 9676.55 ^a^	124,655.40 ± 7703.28 ^b^	75,106.54 ± 8390.90 ^c^
6	sebacic acid	102.08 ± 38.01 ^c^	185.96 ± 21.47 ^b^	330.06 ± 7.81 ^a^
7	benzenepropanoic acid	126,653.60 ± 4870.57 ^c^	232,939.50 ± 13,696.37 ^b^	308,467.40 ± 14,065.29 ^a^
8	3-hydroxymethylglutaric acid	953.71 ± 334.94 ^c^	44,275.26 ± 4401.81 ^a^	28,588.88 ± 2508.37 ^b^
9	pantothenic acid	18,576.24 ± 2409.51 ^b^	30,732.58 ± 729.44 ^a^	28,573.42 ± 1442.77 ^a^
10	maleic acid	15,812.03 ± 530.07 ^b^	19,138.37 ± 1885.22 ^a^	ND
11	gallic acid	ND	ND	953.60 ± 70.65 ^a^
12	azelaic acid	514.28 ± 74.04 ^b^	2086.89 ± 456.11 ^a^	2499.04 ± 81.95 ^a^
13	carnosic acid	255.85 ± 97.86 ^a^	154.00 ± 35.10 ^bc^	101.59 ± 10.50 ^c^
14	suberic acid	526.45 ± 78.84 ^c^	949.76 ± 69.49 ^b^	1140.61 ± 14.77 ^a^
15	taurine	104.42 ± 22.22 ^b^	134.49 ± 9.20 ^b^	241.98 ± 46.64 ^a^
16	3-D-hydroxybutyric acid	N/A	21,610.14 ± 1140.13 ^a^	12,569.20 ± 1097.19 ^b^
17	3-phenyllactic acid	323.82 ± 13.19 ^c^	45,644.99 ± 3154.74 ^b^	80,754.99 ± 2471.99 ^a^
18	ferulic acid	164.00 ± 14.32 ^b^	68.47 ± 14.83 ^c^	389.66 ± 8.26 ^a^
19	benzoic acid	15,910.87 ± 618.60 ^b^	17,915.81 ± 1363.53 ^ab^	19,695.75 ± 1406.25 ^a^
20	pyruvic acid	8210.17 ± 2707.59 ^c^	60,629.57 ± 4063.87 ^b^	142,265.40 ± 11,129.62 ^a^
21	trans-aconitic acid	22,063.48 ± 925.84 ^c^	366,802.50 ± 36,293.39 ^a^	92,576.45 ± 2875.33 ^b^
22	shikimic acid	44,121.80 ± 1832.44 ^c^	510,069.90 ± 66,850.45 ^a^	168,312.90 ± 25,648.89 ^b^
23	2-hydroxyphenylacetic acid	ND	386.09 ± 42.08 ^a^	283.07 ± 39.25 ^b^
24	4-hydroxyphenylacetic acid	ND	2720.14 ± 752.86 ^a^	2473.40 ± 604.67 ^a^
25	lactic acid	ND	118,007.40 ± 25,883.45 ^b^	773,402.90 ± 109,088.20 ^a^
26	methylmalonic acid	ND	186,587.50 ± 29,668.81 ^b^	417,652.30 ± 14,674.55 ^a^
27	succinic acid	20,692.94 ± 1298.51 ^c^	1,837,087.00 ± 163,834.90 ^b^	3,507,057.00 ± 273,419.10 ^a^
28	2-hydroxyisovaleric acid	ND	212,824.20 ± 9026.31 ^b^	252,729.60 ± 9042.65 ^a^
29	glutaric acid	ND	11,660.18 ± 999.07 ^a^	11,354.68 ± 774.53 ^a^
30	oxoglutaric acid	ND	1,420,477.00 ± 37,575.48 ^b^	2,229,738.00 ± 85,047.92 ^a^
31	fumaric acid	3240.64 ± 1114.45 ^b^	43,713.25 ± 7401.76 ^a^	3220.01 ± 510.35 ^b^
32	cis-aconitic acid	8168.66 ± 631.68 ^c^	328,301.50 ± 17,053.28 ^a^	94,821.79 ± 13,381.09 ^b^
33	salicylic acid	155.31 ± 35.43 ^b^	397.64 ± 66.06 ^a^	241.07 ± 23.81 ^b^
34	4-hydroxybenzoic acid	ND	1903.82 ± 130.99 ^a^	1061.65 ± 35.59 ^b^
35	hydroxyphenyllactic acid	ND	8793.06 ± 642.13 ^b^	56,710.12 ± 3942.36 ^a^
36	5-hydroxymethyl-2-furoic acid	1806.40 ± 148.75 ^b^	2365.91 ± 132.96 ^a^	2686.42 ± 171.17 ^a^
37	L-malic acid	586,866.50 ± 30,264.33 ^b^	926,080.00 ± 52,625.91 ^a^	28,759.63 ± 21,314.60 ^c^
38	tartaric acid	14,316.41 ± 1666.98 ^c^	33,865.79 ± 1159.08 ^b^	43,513.52 ± 1592.08 ^a^

Values are mean ± standard deviation (SD) of triplicate measurements. CY denotes non-fermented Chinese yam juice; SP, juice fermented with *Saccharomyces bayanus*; and LB, juice fermented with *Lactobacillus brevis*. ND indicates “Not Detected.” Different superscript letters denote statistically significant differences between samples (*p* < 0.05) as determined by one-way ANOVA.

## Data Availability

The original contributions presented in the study are included in the article/Supplementary Material, further inquiries can be directed to the corresponding author.

## Supplementary Materials

The following supporting information can be downloaded at: https://www.mdpi.com/article/10.3390/foods15061055/s1, Figure S1: Selected (20) non-targeted metabolites in Chinese yam juice samples: CY-SP (A); CY-LB (B); and SP-LB (C). Where CY is control, non-fermented Chinese yam juice, SP is fermented with Saccharomyces bayanus, and LB is sequentially fermented with Lactobacilus brevis. Data represent means ± SD of triplicate readings. Letters indicate statistical differences (*p* < 0.05) between samples based on one-way ANOVA. Figure S2: Changes in total sugar content (mg/mL) of Chinese yam juice. Where CY is control; non-fermented Chinese yam juice, SP is fermented with Saccharomyces bayanus, and LB is sequentially fermented with Lactobacilus brevis. Data represent means ± SD of triplicate readings. Letters indicate statistical differences (*p* < 0.05) between samples based on one-way ANOVA. Table S1: Differential metabolites in juice before and after fermentation (CY-SP). Table S2: Differential metabolites in juice before and after fermentation (CY-LB). Table S3: Differential metabolites in juice before and after fermentation (SP-LB).
