# Supplementary material for: Effects of Sequential Fermentation with Saccharomyces bayanus and Lactobacillus brevis on the Metabolite Composition and Antioxidant Activity of Chinese Yam Juice
Source: Foods. 2026 Mar 17;15(6):1055. doi: 10.3390/foods15061055 (PMC13024804; doi:10.3390/foods15061055)
Supplement: Supplementary file 1 [file foods-15-01055-s001.zip › foods-4159971-supplementary.pdf]

Supplementary materials

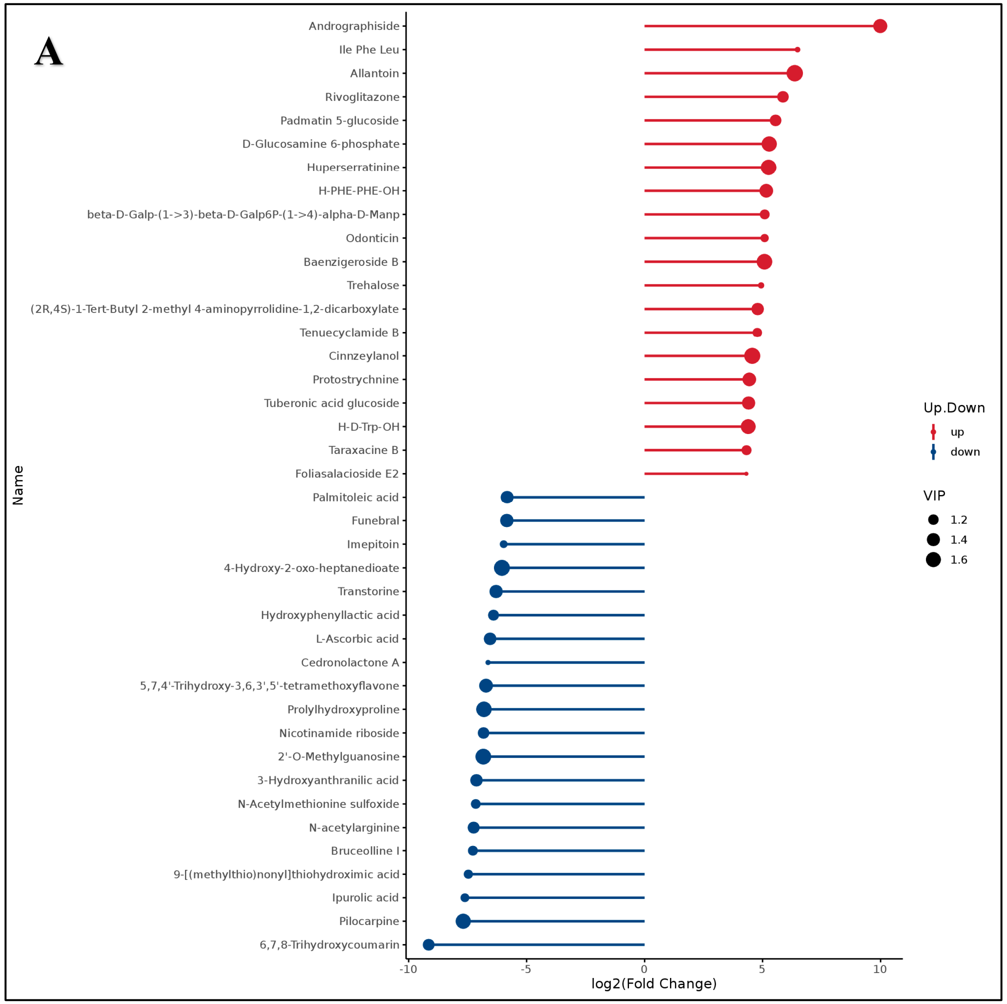

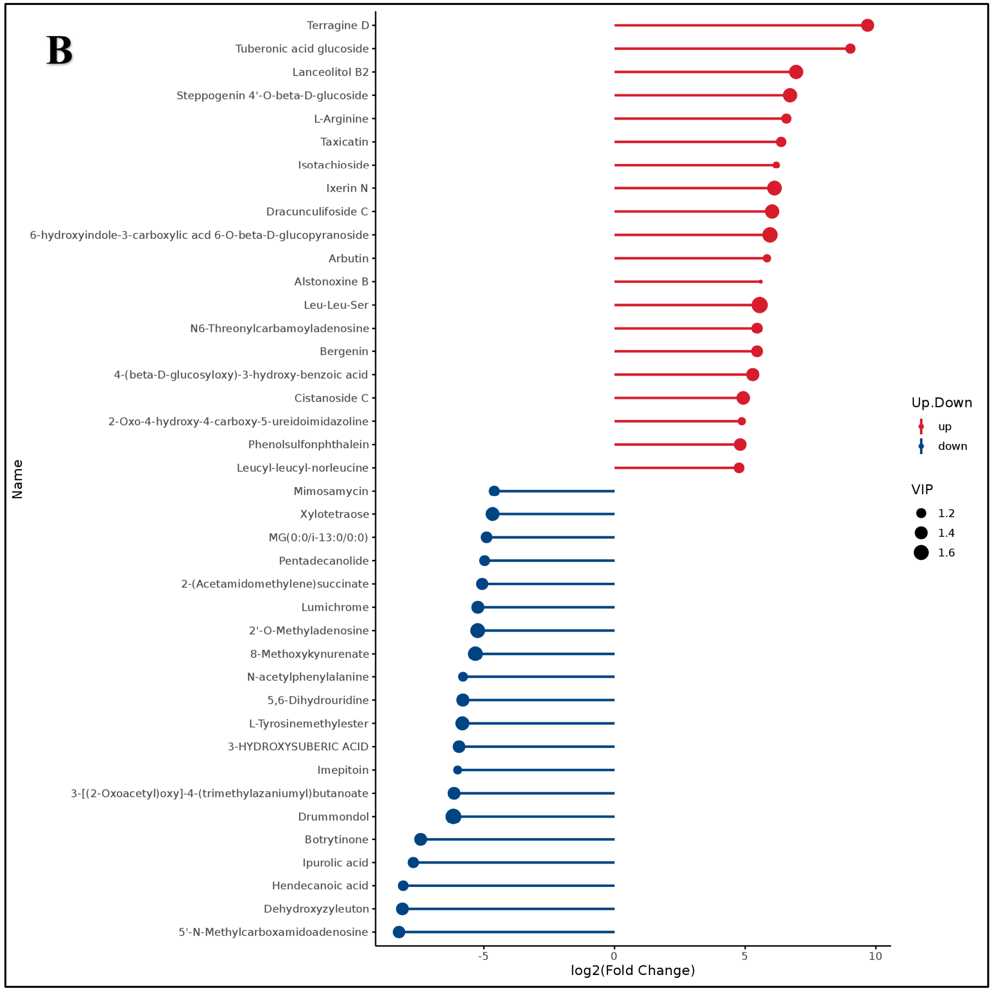

B
Terragine D
Tuberonic acid glucoside
Lanceolitol B2
Steppogenin 4'-O-beta-D-glucoside
L-Arginine
Taxicatin
Isotachioside
Ixerin N
Dracunculifoside C
6-hydroxyindole-3-carboxylic acd 6-O-beta-D-glucopyranoside
Arbutin
Alstonoxine B
Leu-Leu-Ser
N6-Threonylcarbamoyladenosine
Bergenin
4-(beta-D-glucosyloxy)-3-hydroxy-benzoic acid
Cistanoside C
2-Oxo-4-hydroxy-4-carboxy-5-ureidoimidazoline
Phenolsulfonphthalein
Leucyl-leucyl-norleucine
Mimosamycin
Xylotetraose
MG(0:0/i-13:0/0:0)
Pentadecanolide
2-(Acetamidomethylene)succinate
Lumichrome
2'-O-Methyladenosine
8-Methoxykynurenate
N-acetylphenylalanine
5,6-Dihydrouridine
L-Tyrosinemethylester
3-HYDROXYSUBERIC ACID
Imepitoin
3-[(2-Oxoacetyl)oxy]-4-(trimethylazaniumyl)butanoate
Drummondol
Botrytinone
Ipurolic acid
Hendecanoic acid
Dehydroxyzyleuton
5'-N-Methylcarboxamidoadenosine
Name
-5
0
5
10
log2(Fold Change)
Up.Down
up
down
VIP
1.2
1.4
1.6

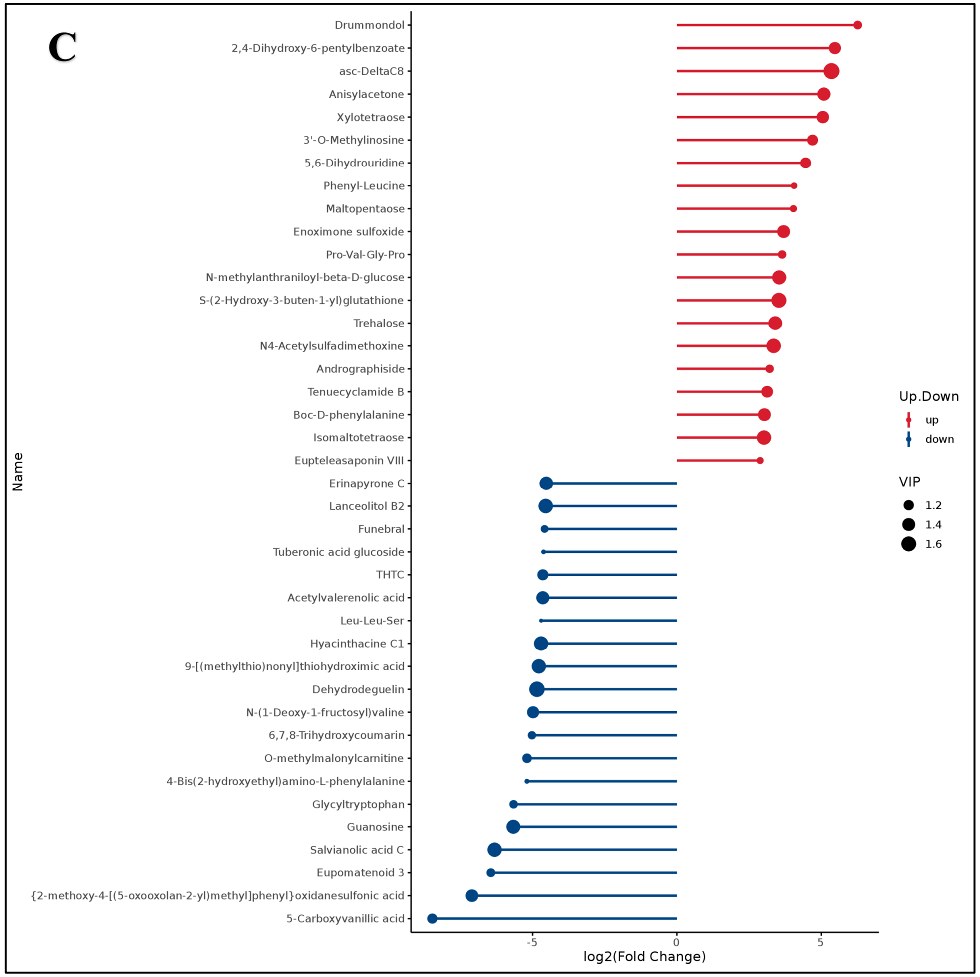

Figure S1. Selected (20) non-targeted metabolites in Chinese yam juice samples: CY-SP (A); CY-LB (B); and SP-LB (C). Where CY is control; non-fermented Chinese yam juice, SP is fermented with *Saccharomyces bayanus*, and LB is sequentially fermented with

Lactobacilus brevis. Data represent means ± SD of triplicate readings. Letters indicate statistical differences ($p < 0.05$) between samples based on one-way ANOVA.

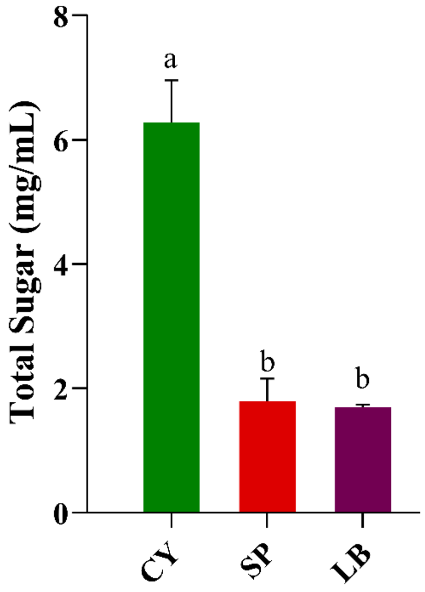

Figure S2. Changes in total sugar content (mg/mL) of Chinese yam juice. Where CY is control; non-fermented Chinese yam juice, SP is fermented with *Saccharomyces bayanus*, and LB is sequentially fermented with *Lactobacilus brevis*. Data represent means ± SD of triplicate readings. Letters indicate statistical differences ($p < 0.05$) between samples based on one-way ANOVA.

Table S1. 1 (CY-SP)

Compound_ID	Name	ClassI	ClassII	FC	VIP	Up.Down
Com_251_neg	Rivoglitazone	Organoheterocyclic compounds	Benzimidazoles	58.244	1.278	up
Com_957_neg	10-Acetylaucubin	Lipids and lipid-like molecules	Prenol lipids	12.073	1.138	up
Com_1128_neg	6-O-Veratroylcatalpol	Phenylpropanoids and polyketides	Tannins	5.949	1.409	up
Com_1644_pos	Humantenine	Alkaloids and derivatives	Gelsemium alkaloids	17.555	1.394	up
Com_832_neg	Phaseoloidin	Organic oxygen compounds	Organooxygen compounds	5.716	1.234	up
Com_715_neg	Tuliposide B	Organic oxygen compounds	Organooxygen compounds	5.766	1.353	up
Com_717_neg	Ethyl (S)-3-hydroxybutyrate glucoside	Lipids and lipid-like molecules	Fatty Acyls	6.288	1.417	up
Com_695_neg	2-Hydroxy-3-(beta-D-xylopyranosyloxy)benzoic acid	Organic oxygen compounds	Organooxygen compounds	2.468	1.507	up
Com_973_neg	cis-3-Hexenyl b-primeveroside	Lipids and lipid-like molecules	Fatty Acyls	6.847	1.411	up
Com_756_neg	3'-O-Methylcatechin	Phenylpropanoids and polyketides	Flavonoids	9.083	1.570	up
Com_958_neg	Tuberonic acid glucoside	Lipids and lipid-like molecules	Fatty Acyls	21.219	1.414	up
Com_1718_pos	Ile Phe Leu	Organic acids and derivatives	Carboxylic acids and derivatives	89.398	1.012	up
Com_149_pos	H-D-Trp-OH	Organoheterocyclic compounds	Indoles and derivatives	20.984	1.590	up
Com_70_neg	(E)-3,4-(Methylenedioxy)cinnamic acid	Phenylpropanoids and polyketides	Cinnamic acids and derivatives	2.641	1.021	up
Com_1483_pos	H-PHE-PHE-OH	Organic acids and derivatives	Carboxylic acids and derivatives	35.670	1.469	up
Com_391_neg	4-Hydroxy-2-oxo-heptanedioate	Organic acids and derivatives	Keto acids and derivatives	0.015	1.710	down
Com_342_pos	Anisomycin	Benzenoids	Phenol ethers	0.025	1.080	down
Com_392_neg	3-HYDROXYSUBERIC ACID	Organic acids and derivatives	Hydroxy acids and derivatives	0.037	1.011	down

Com_169_pos	Prolylhydroxyproline	Organic acids and derivatives	Carboxylic acids and derivatives	0.009	1.655	down
Com_840_neg	9,10,18-Trihydroxyoctadecanoic acid	Lipids and lipid-like molecules	Fatty Acyls	0.345	1.394	down
Com_597_neg	Ipurolic acid	Lipids and lipid-like molecules	Fatty Acyls	0.005	1.110	down
Com_303_pos	Palmitoleic acid	Lipids and lipid-like molecules	Fatty Acyls	0.018	1.384	down
Com_1235_pos	Melisemine	Organoheterocyclic compounds	Quinolines and derivatives	0.057	1.445	down
Com_1038_pos	9-[(methylthio)nonyl]thiohydroximic acid	Organosulfur compounds	Thioamides	0.006	1.128	down
Com_53_neg	Hydroxyphenyllactic acid	Phenylpropanoids and polyketides	Phenylpropanoic acids	0.012	1.229	down
Com_44_neg	L-Ascorbic acid	Organoheterocyclic compounds	Dihydrofurans	0.011	1.353	down
Com_47_pos	3-Hydroxyanthranilic acid	Benzenoids	Benzene and substituted derivatives	0.007	1.341	down
Com_615_neg	Dinex	Benzenoids	Benzene and substituted derivatives	0.415	1.838	down
Com_1804_pos	Chapelieric acid	Organoheterocyclic compounds	Benzopyrans	0.111	1.364	down
Com_959_pos	Aniracetam	Benzenoids	Benzene and substituted derivatives	0.022	1.689	down

CY: Unfermented Chinese yam juice; SP: fermented Chinese yam juice with yeast strain (*Saccharomyces bayanus*)

Table S2. 2 (CY-LB)

Compound_ID	Name	ClassI	ClassII	FC	VIP	Up.Down
Com_901_neg	trans-p-Feruloyl-beta-D-glucopyranoside	Phenylpropanoids and polyketides	Cinnamic acids and derivatives	4.536	1.478	up
Com_964_neg	Glu-Gly-Trp	Organic acids and derivatives	Carboxylic acids and derivatives	8.039	1.250	up
Com_253_neg	Gardenoside	Lipids and lipid-like molecules	Prenol lipids	9.230	1.230	up
Com_70_neg	(E)-3,4-(Methylenedioxy)cinnamic acid	Phenylpropanoids and polyketides	Cinnamic acids and derivatives	3.413	1.236	up
Com_292_neg	Vicenin 2	Phenylpropanoids and polyketides	Flavonoids	4.260	1.596	up
Com_1136_neg	Ixerin N	Lipids and lipid-like molecules	Prenol lipids	69.557	1.572	up
Com_1110_pos	TRIPHENYLMETHANE	Benzenoids	Triphenyl compounds	14.441	1.221	up
Com_832_neg	Phaseoloidin	Organic oxygen compounds	Organooxygen compounds	6.906	1.209	up
Com_1034_neg	Spicatocadinanolide A	Lipids and lipid-like molecules	Prenol lipids	2.526	1.204	up
Com_1067_neg	Saccharumoside C	Organic oxygen compounds	Organooxygen compounds	2.541	1.222	up
Com_1483_pos	H-PHE-PHE-OH	Organic acids and derivatives	Carboxylic acids and derivatives	16.994	1.067	up
Com_553_neg	Cladobotrin III	Organoheterocyclic compounds	Pyrans	3.447	1.419	up
Com_1143_neg	Seguinoside E	Phenylpropanoids and polyketides	Tannins	2.677	1.753	up
Com_1019_neg	Deacetylasperulosidic acid ethyl ester	Lipids and lipid-like molecules	Prenol lipids	5.014	1.683	up
Com_748_neg	Isotachioside	Organic oxygen compounds	Organooxygen compounds	72.935	1.089	up
Com_23_neg	1,5-Isoquinolinediol	Organoheterocyclic compounds	Isoquinolines and derivatives	0.044	1.185	down

Com_957_pos	8-Methoxykynurenate	Organoheterocyclic compounds	Quinolines and derivatives	0.025	1.590	down
Com_1026_pos	Mimosamycin	Organoheterocyclic compounds	Isoquinolines and derivatives	0.041	1.263	down
Com_392_neg	3-HYDROXYSUBERIC ACID	Organic acids and derivatives	Hydroxy acids and derivatives	0.016	1.378	down
Com_309_neg	Botrytinone	Organic oxygen compounds	Organooxygen compounds	0.006	1.399	down
Com_597_neg	Ipurolic acid	Lipids and lipid-like molecules	Fatty Acyls	0.005	1.279	down
Com_1510_pos	Nigerapyrone B	Benzenoids	Benzene and substituted derivatives	0.099	1.044	down
Com_111_neg	Hendecanoic acid	Lipids and lipid-like molecules	Fatty Acyls	0.004	1.241	down
Com_373_pos	Aspartylphenylalanine	Organic acids and derivatives	Carboxylic acids and derivatives	0.460	1.227	down
Com_506_neg	Drummondol	Organoheterocyclic compounds	Oxepanes	0.014	1.692	down
Com_613_neg	Boc-D-phenylalanine	Organic acids and derivatives	Carboxylic acids and derivatives	0.126	1.634	down
Com_247_pos	Ambroxide	Organoheterocyclic compounds	Naphthofurans	0.118	1.787	down
Com_376_pos	2'-O-Methyladenosine	Nucleosides, nucleotides, and analogues	Purine nucleosides	0.026	1.584	down
Com_454_pos	Hexadecanedioic acid	Lipids and lipid-like molecules	Fatty Acyls	0.121	1.074	down
Com_980_pos	cspyrone B1	Organic oxygen compounds	Organooxygen compounds	0.237	1.095	down

CY: Unfermented Chinese yam juice LB: fermented Chinese yam juice with bacterial strain (*Lactobacillus brevis*)

Table S3 3 (SP-LB)

Compound_ID	Name	ClassI	ClassII	FC	VIP	Up.Down
Com_704_neg	asc-DeltaC8	Organic oxygen compounds	Organooxygen compounds	40.86	1.725	up
Com_266_pos	Lumichrome	Organoheterocyclic compounds	Pteridines and derivatives	2.39	1.326	up
Com_93_neg	Anisylacetone	Benzenoids	Phenol ethers	34.05	1.430	up
Com_441_neg	2,4-Dihydroxy-6-pentylbenzoate	Benzenoids	Benzene and substituted derivatives	44.35	1.333	up
Com_613_neg	Boc-D-phenylalanine	Organic acids and derivatives	Carboxylic acids and derivatives	8.17	1.396	up
Com_1000_neg	Daumone 4	Organic oxygen compounds	Organooxygen compounds	2.22	1.399	up
Com_1365_pos	Dianthoside	Organic oxygen compounds	Organooxygen compounds	2.74	1.084	up
Com_488_neg	Carboxyibuprofen	Phenylpropanoids and polyketides	Phenylpropanoic acids	5.75	1.434	up
Com_471_neg	isobutyryl carnitine	Lipids and lipid-like molecules	Fatty Acyls	3.80	1.187	up
Com_677_neg	4-Hydroxyphenylacetylglutamic acid	Organic acids and derivatives	Carboxylic acids and derivatives	1.72	1.363	up
Com_22_neg	N-Formyl-L-aspartate	Organic acids and derivatives	Carboxylic acids and derivatives	2.82	1.454	up
Com_653_neg	Itoside M	Organic oxygen compounds	Organooxygen compounds	2.09	1.268	up
Com_1167_neg	Henryoside	Organic oxygen compounds	Organooxygen compounds	1.70	1.300	up
Com_295_neg	Isomaltotetraose	Organic oxygen compounds	Organooxygen compounds	8.09	1.548	up
Com_217_neg	Trehalose	Organic oxygen compounds	Organooxygen compounds	10.60	1.498	up
Com_375_neg	Erinapyrone C	Organoheterocyclic compounds	Pyrans	0.04	1.459	down

Com_1486_pos	Neoamphimedine	Organoheterocyclic compounds	Quinolines and derivatives	0.13	1.125	down
Com_1683_pos	Cedronolactone A	Lipids and lipid-like molecules	Prenol lipids	0.12	1.247	down
Com_928_pos	5-Carboxyvanillic acid	Benzenoids	Benzene and substituted derivatives	0.00	1.191	down
Com_405_pos	5'-Methylthioadenosine	Nucleosides, nucleotides, and analogues	5'-deoxyribonucleosides	0.17	1.200	down
Com_576_pos	Cortisone acetate	Lipids and lipid-like molecules	Steroids and steroid derivatives	0.08	1.517	down
Com_1229_pos	L-phenylalanyl-L-proline	Organic acids and derivatives	Carboxylic acids and derivatives	0.07	1.127	down
Com_1136_neg	Ixerin N	Lipids and lipid-like molecules	Prenol lipids	0.11	1.288	down
Com_376_pos	2'-O-Methyladenosine	Nucleosides, nucleotides, and analogues	Purine nucleosides	0.19	1.545	down
Com_1221_pos	Glycyltryptophan	Organic acids and derivatives	Carboxylic acids and derivatives	0.02	1.103	down
Com_314_pos	Biotin sulfone	Organoheterocyclic compounds	Biotin and derivatives	0.06	1.415	down
Com_1388_pos	Acetylvalerenolic acid	Lipids and lipid-like molecules	Prenol lipids	0.04	1.422	down
Com_546_pos	Epirosmanol	Lipids and lipid-like molecules	Prenol lipids	0.05	1.399	down
Com_1386_pos	Eupomatenoid 3	Phenylpropanoids and polyketides	2-arylbenzofuran flavonoids	0.01	1.115	down
Com_740_neg	Pseudolaroside A	Organic oxygen compounds	Organooxygen compounds	0.24	1.039	down

LB: fermented Chinese yam juice with bacterial strain (*Lactobacillus brevis*); SP: fermented Chinese yam juice with yeast strain (*Saccharomyces bayanus*)
